# Inferior ST segment elevation myocardial infarction combined with hyperextension cervical spine injury: A rare case report

**DOI:** 10.1097/MD.0000000000033408

**Published:** 2023-03-31

**Authors:** Jiaying Li, Ping Zhong, Zheng Wang, Shufang Han

**Affiliations:** a Weifang Medical University, Weifang, Shandong Province, China; b Department of Cadre Ward, The 960th Hospital of the Joint Service Support Force of the People's Liberation Army, Jinan, Shandong Province, China; c Department of Cardiology, The 960th Hospital of the Joint Service Support Force of the People's Liberation Army, Jinan, Shandong Province, China.

**Keywords:** hyperextension cervical spine injury, left circumflex coronary artery, rehabilitation, ST-segment elevation myocardial infarction, thrombus

## Abstract

Inferior ST-segment elevation myocardial infarction (STEMI) is usually caused by acute occlusion of the right coronary artery or left circumflex coronary artery (LCX). Inferior STEMI can cause a high-grade atrioventricular block, sinus bradycardia, and hypotension, leading to hemodynamic collapse, syncope, and disturbance of consciousness.

**Patient concerns:**

A case of a sudden disturbance of consciousness after chest tightness as the initial symptom, followed by incomplete paralysis and paresthesia of the extremities due to the collision of the face with the ground.

**Diagnoses:**

Coronary angiography showed about 99% of stenosis in the LCX. Cervical spine magnetic resonance imaging showed C2/3, C3/4, C4/5, and C5/6 intervertebral disc herniation with secondary spinal stenosis, spinal cord compression, and edema. The patient was diagnosed with inferior STEMI combined with hyperextension cervical spine injury.

**Interventions and outcomes:**

Bivalirudin was used for anticoagulation, the LCX lesion was pre-expanded with a balloon and the thrombus was removed, and anti-platelet aggregation therapy was given postoperatively. After rehabilitation therapy, hyperextension cervical spine injury improved. There was no recurrence of syncope and precordial pain during the 6-month follow-up.

**Lessons:**

Hyperextension cervical spine injury has unique hemodynamic features that mimic those associated with inferior STEMI, so a detailed medical history inquiry and physical examination should be carried out to avoid missed diagnoses.

## 1. Introduction

Inferior ST-segment elevation myocardial infarction (STEMI) is caused by the occlusion of the right coronary artery or left circumflex coronary artery (LCX), especially when involving the right ventricle, may have pronounced hemodynamic complications associated with increased risk of death, shock, and arrhythmias.^[1]^ The important changes in electrocardiogram (ECG) and their relationship with the infarct-related artery were recognized in the 1980s, and several ECG criteria have been proposed to identify inferior STEMI.^[2,3]^ Syncope is a transient disturbance of consciousness caused by insufficient cerebral perfusion, with sudden onset but full recovery, and inferior STEMI is an important cause of syncope.

The cervical spine consists of 7 vertebrae, which support the head and its movements, protect the spinal cord, and supply blood to the brain. The cervical spine is most vulnerable to injury due to its over-reliance on ligamentous structures for stability. Traumatic central cord syndrome is the most commonly encountered type of spinal cord injury, manifested as incomplete quadriplegia and sensory dissociation, which is mainly caused by hyperextension cervical spine injury in patients >50 years with a narrow spinal canal.^[4,5]^ Here, we reported a case with inferior STEMI combined with hyperextension cervical spine injury, the male patient with a sudden disturbance of consciousness after chest tightness as the initial symptom, followed by incomplete paralysis and paresthesia of the extremities due to the collision of the face with the ground. This case is rare in clinical practice and is easily misdiagnosed as cerebrovascular diseases to miss the best time to save spinal nerve function. The diagnosis and treatment processes were as follows:

## 2. Case report

A 70-year-old man with a 3-year history of diabetes was admitted to our hospital for treatment due to sudden chest tightness and disturbance of consciousness for about 10 seconds, and fainting to the ground 13 hours ago. No dizziness, headache, nausea, vomiting, and limb convulsions at the time of onset. The patient was in a passive prone position on admission, with both upper extremities pressed against the chest. Routine examination showed that the blood pressure was 61/33 mm Hg, and the heart rate was 54 beats/min. The patient was conscious, the left zygomatic face, cheeks, and mandibular skin were bruised and swollen, and the periorbital area of the right eye was bruised and bleeding. Touching the skin of both upper limbs showed pain, and touching the skin of the nipple showed hyperalgesia. The muscle strength of the right upper extremity was grade 0, the left upper extremity was grade III, the left lower extremity was grade III to IV, and the right lower extremity was grade II to III. The Achilles tendon reflex was hyperreflexia on both sides, the Hoffman sign was positive on both hands, and the Barthel sign was positive on both sides.

**Figure 1.**
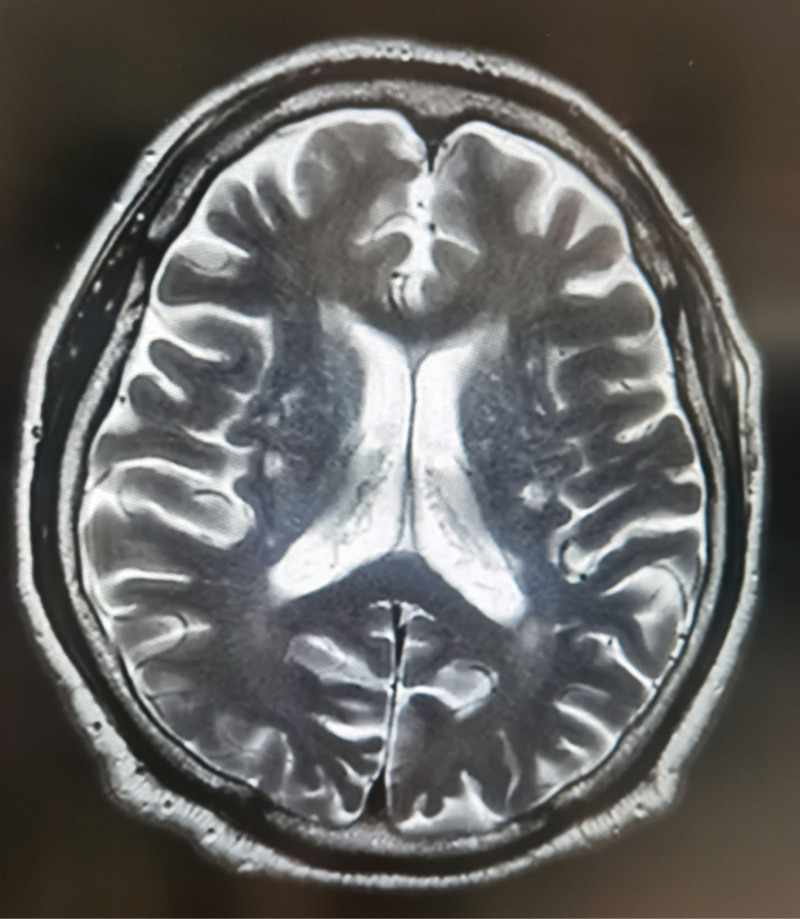
The image of brain magnetic resonance imaging (MRI). The patient showed multiple lacunar infarcts and brain atrophy, consistent with the manifestations of cerebral arteriosclerosis, and acute cerebral infarction was excluded.

**Figure 2.**
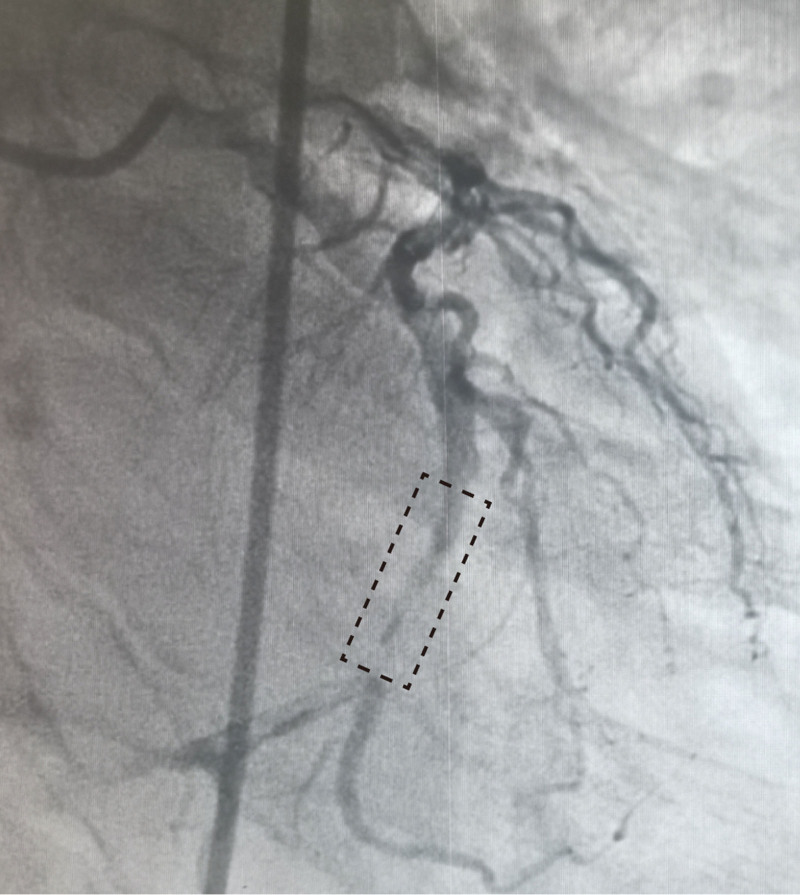
The image of coronary angiogram preoperative. The left circumflex coronary artery showed about 99% of stenosis (black dotted frame).

The 12-lead ECG showed sinus rhythm and an upward oblique elevation of the ST segment in leads II, III, and aVF. Biochemical tests showed Troponin I > 50 μg/L (reference: 0–0.034 μg/L) and n-terminal pro-brain natriuretic peptide (NT-proBNP) at 1271 ng/L (reference: 0–125 ng/L). Brain magnetic resonance imaging (MRI) revealed multiple lacunar infarcts and brain atrophy, consistent with the manifestations of cerebral arteriosclerosis, and acute cerebral infarction was excluded (Fig. 1). Coronary angiography showed about 75% of stenosis in left anterior descending coronary artery, about 99% of stenosis in LCX, obvious thrombus shadow distal to the left main branch, and about 60% of stenosis in proximal right coronary artery (Fig. 2). The patient’s examination results were consistent with the diagnosis of inferior STEMI. This study was approved by the Ethics Committee of the The 960th Hospital of the Joint Service Support Force of the People's Liberation Army.

The patient was given Bivalirudin for anticoagulation treatment, and the LCX lesion was pre-dilated with a balloon. Subsequently, an extractor suction catheter was used to repeatedly aspirate the lesion, and a large amount of thrombus was sucked out, but stenostic LCX is still not visualized after coronary angiography. After an intracoronary injection of 12 mL of Tirofiban injection, the blood flow was not restored. Therefore, 150,000 units of Urokinase were administered into the microcatheter for thrombolysis, and the proximal and middle LCX was visualized after coronary angiography, but the distal LCX was not clearly visualized. The patients were given intensive anti-platelet aggregation therapy after surgery.

**Figure 3.**
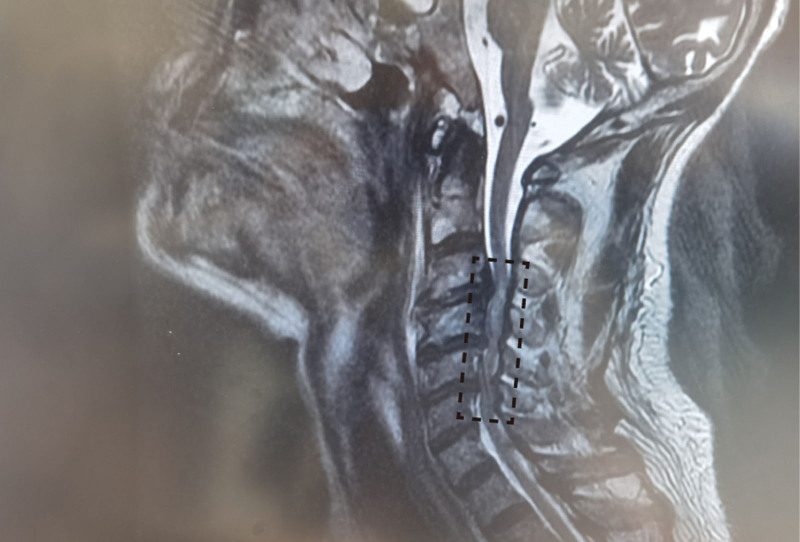
The image of cervical spine MRI. The patient showed C2/3, C3/4, C4/5, and C5/6 intervertebral disc herniation with secondary spinal stenosis (black dotted frame), accompanied by spinal cord compression and edema. MRI = magnetic resonance imaging.

The patient’s facial hematoma hemorrhage worsened postoperatively, Bivalirudin was immediately discontinued, local cold compresses were bandaged, and 0.15 μg/kg/min of tirofiban was continuously intravenous injected for 3 days. After 2 days of hemodynamic stabilization, the cervical spine MRI examination showed the following results: cervical spine degeneration; C2/3, C3/4, C4/5, and C5/6 intervertebral disc herniation with secondary spinal stenosis; cervical cord compression and edema (Fig. 3). The patient’s examination results were consistent with the diagnosis of hyperextension cervical spine injury.

**Figure 4.**
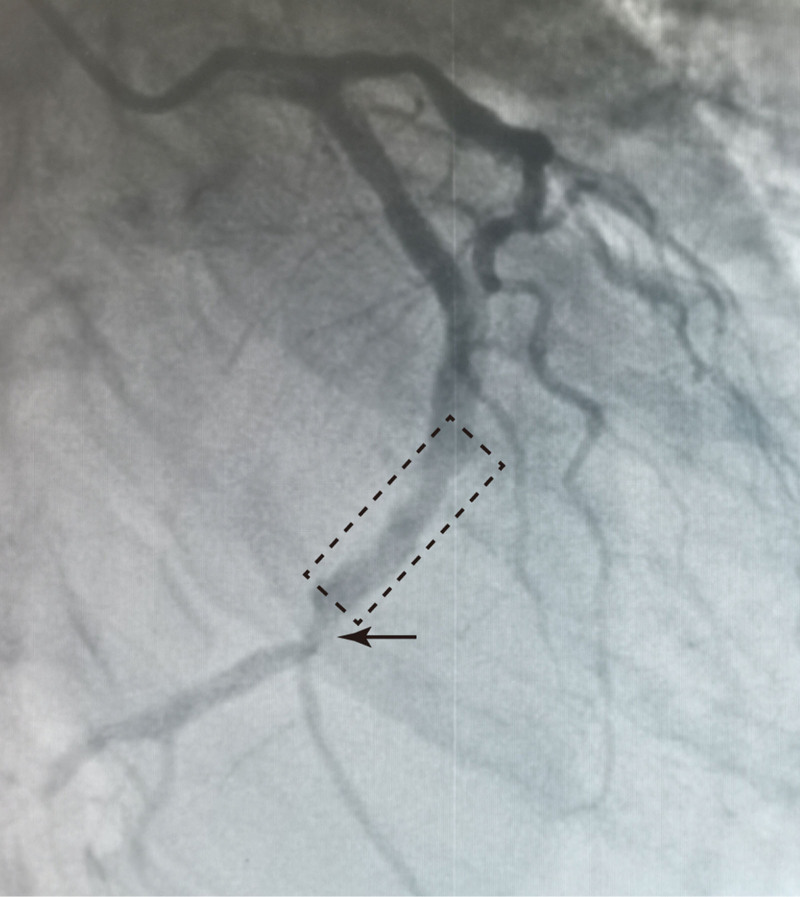
The image of coronary angiogram at 3 weeks postoperative. The local lumen of the left circumflex coronary artery showed about 75% of stenosis (black arrow), and no obvious restenosis at the balloon dilation (black dotted frame).

Because the patient had just undergone percutaneous coronary intervention (PCI), cervical decompression and internal fixation cannot be performed immediately, which will lead to the risk of serious complications, such as cardiac arrest, malignant arrhythmia, and cardiac rupture. Therefore, the patient was immobilized with a cervical collar and given conservative treatment with an appropriate amount of Aspirin, Ticagrelor, and Atorvastatin. On the 5th day after the operation, echocardiography revealed 37% of left ventricular ejection fraction, segmental abnormal motion of the left ventricular wall (inferior and posterior wall), ventricular septal thickening, mitral regurgitation (mild), and decreased left ventricular systolic and diastolic function. One week later, the muscle strength of the lower extremity gradually improved. Coronary angiography was repeated after anti-platelet aggregation treatment for 3 weeks, and found about 75% of stenosis in the left anterior descending coronary artery, about 75% of stenosis in the local lumen of LCX, and no obvious restenosis at the balloon dilation (Fig. 4). After 4 weeks of treatment, the muscle strength of the upper extremity had no significant improvement; however, the muscle strength of the left lower extremity recovered to grade IV and the muscle strength of the right lower extremity recovered to grade III. The patient could sit up independently and stand by the sickbed with the help of family members. The patient no longer had symptoms of chest tightness and chest pain. The patient continued to be given medication and rehabilitation after discharge. During the 6-month follow-up, the patient had no recurrence of syncope and precordial pain.

## 3. Discussion

According to epidemiological statistics, syncope accounts for 1 to 3% of emergency hospital admission. Syncope is clinically divided into 4 types, including reflex syncope, orthostatic hypotension syncope, and cardiogenic syncope.^[6]^ Among them, cardiogenic syncope is the most dangerous, mainly due to various arrhythmias and organic cardiovascular diseases. Increasing literature indicated that patients with inferior STEMI or aortic stenosis can trigger or induce reflex syncope.^[7]^ In clinical practice, physicians are required to quickly and accurately identify the cause of syncope and perform risk stratification based on the patient’s clinical characteristics and related examinations, so as to optimize the syncope treatment process. In this case, the male patient had chest tightness and sudden disturbance of consciousness, and syncope occurred when changing from sitting to standing position. Therefore, it was considered that cardiogenic syncope combined with reflex syncope is highly likely.

PCI is the most effective treatment for patients with inferior STEMI.^[8]^ About 20% of inferior patients undergoing PCI are at high risk of major bleeding, which is a stronger predictor of death than myocardial infarction perioperative.^[9]^ Bivalirudin, as a synthetic direct thrombin inhibitor, can effectively prolong blood coagulation time and exert an anticoagulant effect to prevent contact thrombosis.^[10]^ Compared with traditional heparin anticoagulant therapy, Bivalirudin is mainly metabolized by the kidneys with a half-life of only about 25 minutes and showed a shorter duration of anticoagulation and a lower incidence of bleeding.^[11]^ Bikdeli et al pooled data from 8 randomized clinical trials to compare the efficacy of Bivalirudin with Heparin and found that Bivalirudin significantly reduces the risk of bleeding events, without increasing adverse clinical events and stent thrombosis events.^[12]^ Based on current guideline consensus, Bivalirudin is recommended for patients at high bleeding risk and for Heparin-induced thrombocytopenia. In this patient, hematoma was obvious in the frontal face and mandible, indicating a high risk of bleeding. Therefore, Bivalirudin was chosen to balance the risk of bleeding and thrombosis.

Coronary angiography showed about 99% stenosis of LCX, and the LCX lesion was pre-expanded with a balloon and the thrombus was removed, and then the proximal and middle LCX was visualized. After the intervention, the patient’s hematoma swelling increased with dyspnea, so Bivalirudin was discontinued, and the hematoma did not worsen after symptomatic treatment. Coronary angiography was repeated at 3 weeks postoperative and found about 75% stenosis in the local lumen of LCX, and no obvious restenosis at the balloon dilation, indicating PCI is successful.

Hyperextension cervical spine injury accounts for about 47 to 65% of various types of cervical spine injury, which is caused by the overextension of the cervical spine, with or without damage to the spine and surrounding blood vessels and soft tissues. The diagnosis of hyperextension cervical spine injury is mainly based on the following: frontal and facial skin trauma with neck pain symptoms; manifested as traumatic central cord syndrome, the upper extremity symptoms are heavier than the lower extremity symptoms, the motor dysfunction is more serious than the sensory dysfunction, decreased muscle strength of the upper extremity, especially hand dysfunction, and sensory dissociation dysfunction; X-ray, computed tomography. and MRI examinations indicate cervical spine injury.^[13]^

In this case, the 70-year-old male patient developed a frontal and facial hematoma after a fall, with incomplete quadriplegia and marked hyperalgesia, the result of cervical spine MRI examination confirmed the diagnosis of hyperextension cervical spine injury. After rehabilitation therapy, the muscle strength of the lower extremity improved. Similarly, Kumagai et al reported a case of syncope after trauma complicated by acute inferior myocardial infarction and diagnosed with neurogenic shock caused by an acute cervical spine injury.^[14]^ The patient is able to walk about 50 m without assistance after 4 months of rehabilitation, but the upper extremity motor ability has only slightly improved.

In conclusion, patients with inferior STEMI usually have syncope or consciousness disturbance following hemodynamic collapse, therefore, acute cervical spine injury caused by falling to the ground can occur together with inferior STEMI. Hyperextension cervical spine injury has unique hemodynamic features that mimic those associated with inferior STEMI but requires very different treatment. For patients with frontal and facial skin trauma combined with incomplete paralysis and paresthesia of the extremities, a detailed medical history inquiry and physical examination should be carried out to identify whether there is a possibility of cervical spine injury. At the same time, improve the relevant auxiliary examinations to make an early diagnosis and avoid the further development of the disease.

## Author contributions

**Conceptualization:** Shufang Han.

**Formal analysis:** Zheng Wang, Shufang Han.

**Investigation:** Jiaying Li, Ping Zhong.

**Methodology:** Zheng Wang.

**Resources:** Jiaying Li, Ping Zhong, Zheng Wang.

**Software:** Jiaying Li.

**Supervision:** Shufang Han.

**Writing – original draft:** Jiaying Li.

**Writing – review & editing:** Shufang Han.
